# Four genomic islands that mark post-1995 pandemic *Vibrio parahaemolyticus *isolates

**DOI:** 10.1186/1471-2164-7-104

**Published:** 2006-05-03

**Authors:** Catherine C Hurley, AnneMarie Quirke, F Jerry Reen, E Fidelma Boyd

**Affiliations:** 1 Department of Microbiology, University College Cork, National University of Ireland, Cork, Ireland

## Abstract

**Background:**

*Vibrio parahaemolyticus *is an aquatic, halophilic, Gram-negative bacterium, first discovered in 1950 in Japan during a food-poisoning outbreak. Infections resulting from consumption of *V. parahaemolyticus *have increased globally in the last 10 years leading to the bacterium's classification as a newly emerging pathogen. In 1996 the first appearance of a pandemic *V. parahaemolyticus *clone occurred, a new O3:K6 serotype strain that has now been identified worldwide as a major cause of seafood-borne gastroenteritis.

**Results:**

We examined the sequenced genome of *V. parahaemolyticus *RIMD2210633, an O3:K6 serotype strain isolated in Japan in 1996, by bioinformatic analyses to uncover genomic islands (GIs) that may play a role in the emergence and pathogenesis of pandemic strains. We identified 7 regions ranging in size from 10 kb to 81 kb that had the characteristics of GIs such as aberrant base composition compared to the core genome, presence of phage-like integrases, flanked by direct repeats and the absence of these regions from closely related species. Molecular analysis of worldwide clinical isolates of *V. parahaemolyticus *recovered over the last 33 years demonstrated that a 24 kb region named *V. parahaemolyticus *island-1 (VPaI-1) encompassing ORFs VP0380 to VP0403 is only present in new O3:K6 and related strains recovered after 1995. We investigated the presence of 3 additional regions, VPaI-4 (VP2131 to VP2144), VPaI-5 (VP2900 to VP2910) and VPaI-6 (VPA1254 to VPA1270) by PCR assays and Southern blot analyses among the same set of *V. parahaemolyticus *isolates. These 3 VPaI regions also gave similar distribution patterns amongst the 41 strains examined.

**Conclusion:**

The 4 VPaI regions examined may represent DNA acquired by the pandemic group of *V. parahaemolyticus *isolates that increased their fitness either in the aquatic environment or in their ability to infect humans.

## Background

*Vibrio parahaemolyticus *is a halophilic, Gram-negative bacterium, first discovered in 1950 during a food poisoning outbreak in Osaka, Japan. *V. parahaemolyticus *is a seafood-borne pathogen, which is a major causative agent of gastroenteritis particularly in regions with high seafood consumption. In Taiwan, Japan, and South East Asian countries, *V. parahaemolyticus *causes over half of all food poisoning outbreaks of bacterial origin [1]. In recent years outbreaks of *V. parahaemolyticus *infection have increased in the United States; and *V. parahaemolyticus *infection is estimated to be responsible for 5000 illnesses per year [2]. *V. parahaemolyticus *infections that caused gastroenteritis up until 1996 were associated with many different serotypes, with a predominance of O4 serogroup strains among clinical samples in the United States [3-5]. In 1996 the first appearance of a pandemic *V. parahaemolyticus *clone occurred, a new O3:K6 serotype strain that has now been identified worldwide as a major cause of seafood-borne gastroenteritis [2-4,6-9].

Clinical isolates of *V. parahaemolyticus *produce two major virulence factors; the thermostable direct hemolysin (TDH) encoded by *tdh*, and TDH-related hemolysin encoded by *trh*. Several studies have demonstrated that most pandemic *V. parahaemolyticus *new O3:K6 serotype isolates contain the *tdh *gene but not the *trh *gene and are hemolytic on Wagatsuma agar designated Kanagawa phenomenon positive strains (KP^+^) [2,10,11]. A number of additional biomarkers have been identified in pandemic *V. parahaemolyticus *serotype O3:K6 isolates; these include a unique *toxRS *sequence, a histone-like DNA-binding protein and an open reading frame VP2905, all found to be present exclusively in these strains [6,12-14]. The recently emerged serotypes O4:K68, O1:KUT, O1:K25 and O1:K41 have been shown to be clonally related to new O3:K6 serotype strains isolated after 1995, all forming a pandemic group [3,6,14,15]. It had been suggested that the new O3:K6 group strains might have emerged as a result of the transfer of genetic elements, and the *V. parahaemolyticus *phage f237 is believed to be responsible for the pandemic potential of *V. parahaemolyticus *[16]. However, many post-1995 *V. parahaemolyticus *isolates lack phage f237 [8,17].

Genomic islands (GIs) are another group of chromosomal regions, which are acquired by horizontal gene transfer that may increase fitness of the bacterium in a particular environment. For example, virulence genes present on pathogenicity islands or genes that provide diverse metabolic capabilities on metabolic islands can play an important role in bacterial survival in diverse environments [18]. The DNA sequences of GIs are compositionally biased from their host genome in terms of G+C content, genome signature (dinucleotide frequency) and codon usage patterns [18]. As well as aberrant DNA composition, GIs have the general characteristics of encoding a bacteriophage-like integrase, are flanked by repeat sequences, and many GIs insert adjacent to tRNA genes probably indicating a similar mechanism of chromosomal integration. GIs are usually present in a subset of strains of a species and absent from closely related species. From the genomic sequence of *V. parahaemolyticus *RIMD2210633, an 81 kb region on chromosome 2 with a G+C content of 39% compared to the overall G+C content for the entire genome of 45% was identified [19]. This potential pathogenicity island encoded a type III secretion (TTS) system, which in other pathogenic bacteria export bacterial proteins directly into host cells. The TTS system on chromosome 2 was shown only to be present among *V. parahaemolyticus *isolates recovered after 1995 [19].

In the present study, we interrogated the complete genome sequence of *V. parahaemolyticus *strain RIMD2210633 an O3:K6 strain clinical isolate by bioinformatic and molecular analyses to identify GIs that may mark pandemic isolates. We uncovered 7 regions, ranging in size from 10 kb to 81 kb with the characteristics of GIs. We named these 7 regions *Vibrio parahaemolyticus *island-1 (VPaI-1) to VPaI-7. We examined the distribution of VPaI-1 among a collection of 41 *V. parahaemolyticus *natural isolates recovered between 1970 and 2003. By molecular analyses, the VPaI-1 region was only present amongst post-1995 serotype O3:K6 and related pandemic isolates indicating that this region may play a role in the emergence of pandemic isolates. In addition, we investigated the distribution of VPaI-4, VPaI-5, and VPaI-6 among the same collection of *V. parahaemolyticus *isolates and all 3 regions gave a similar distribution as VPaI-1 indicating that these regions, which encoded a number of potential virulence genes, may be involved in the emergence of pandemic isolates.

## Results and discussion

### Genomic islands (GIs) identified in *V. parahaemolyticus *RIMD2210633

We examined the whole genome sequence of *V. parahaemolyticus *RIMD2210633 to identify GIs that could potentially mark this pandemic clone. Initially we identified 9 regions of greater than 10 kb with aberrant G+C content that encoded integrase or transposase genes. These 9 regions included a prophage and an integron as well as 7 additional regions, which we named *V. parahaemolyticus *island-1 (VPaI-1) to VPaI-7. The 7 genomic islands (GIs) ranged in size from 10 kb to 81 kb, were flanked by direct repeats, and 6 of the 7 GIs had a G+C content lower (ranging from 38% to 43%) than the overall genome G+C content of 45% (Table 1). All GIs encoded an integrase gene with the exception of VPaI-7, which contained a number of transposase genes. VPaI-7 is an 81 kb region that encoded a TTS system and the tdh gene and was previously identified as a potential pathogenicity island (Table 1) [19]. Five GI regions were present on chromosome 1 and two on chromosome 2. Four of the GIs identified inserted adjacent to tRNA genes; VPaI-1 (tRNA-Met), VPaI-2 (tmRNA), VPaI-3 (tRNA-Ser) and VPaI-4 (tRNA-Ser) (Table 1). Using the program developed by van Passel and co-workers, we examined the compositional dissimilarities among the 7 VPaI regions identified compared to the host genome (Table 1) [20,21]. A high genomic dissimilarity (δ*) between a VPaI region and the *V. parahaemolyticus *genome sequence indicates a heterologous origin of the GI. All 7 VPaI regions that we examined had high δ* values compared to the average for the genome of 38 for chromosome 1 and 36 for chromosome 2 (window size 10 kb) (Table 1). In addition, we investigated the percent of genomic fragments of sizes equal to each VPaI with lower δ* and between 94 and 100 percent of genomic fragments examined had a lower δ* than the GI examined indicating that the 7 VPaI regions uncovered had an aberrant base composition compared to the rest of the genome (Table 1).

### Comparative analysis

**Figure 1:**
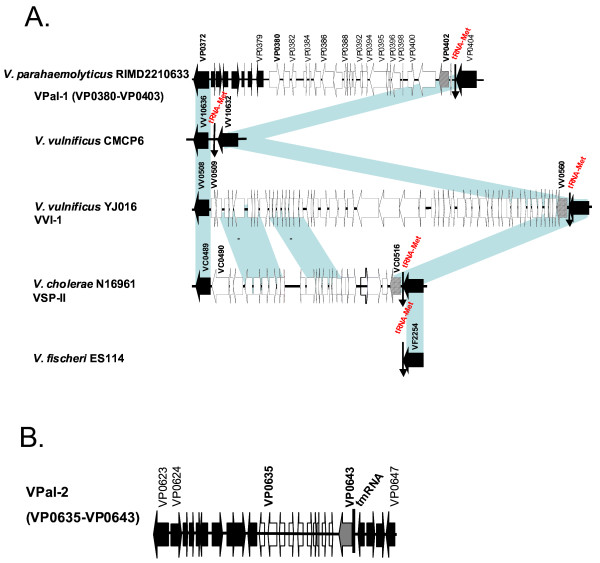
**A. Schematic representation of *V. parahaemolyticus *island-1 (VPaI-1) and the homologous tRNA-Met insertion site in *V. vulnificus *strains YJ016 and CMCP6, *V. cholerae *N16961, and *V. fischeri *ES114**. Block arrows represent annotated open reading frames (ORFs). Numbers above block arrows indicate ORF number on the sequenced genome strain. Black filled in ORFs represent core chromosomal genes. Open arrows represent genomic island ORFs. Shaded arrows represent integrase genes. Vertical arrows represent homologous tRNA-Met genes within each genome. Shaded blocks indicate homologous ORFs among the *Vibrio *species. B. Schematic representation of VPaI-2 from *V. parahaemolyticus *RIMD2210633.

**Figure 2:**
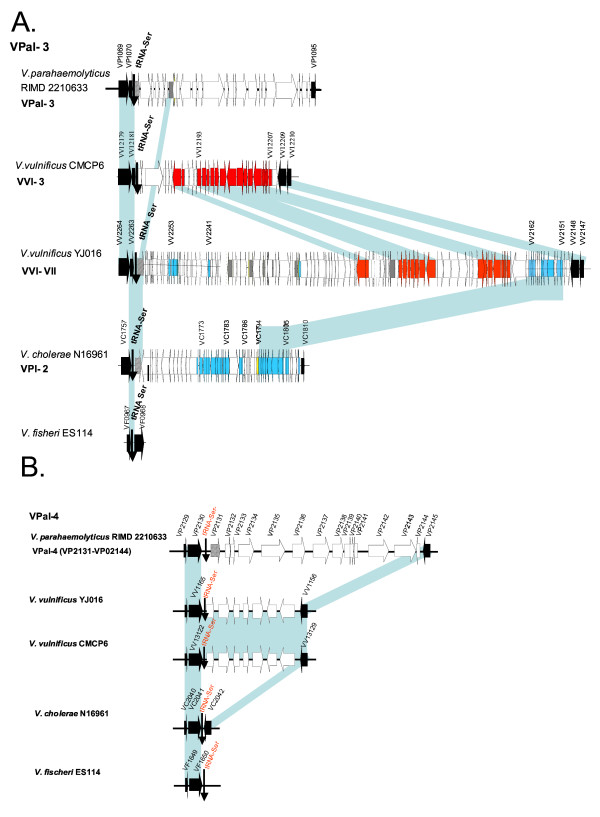
**A and B. Schematic representation of *V. parahaemolyticus *island-3 (VPaI-3) and VPaI-4, and the homologous insertion site among the 4 sequenced *Vibrio *species**. Block arrows represent annotated ORFs. Black filled in ORFs represent core chromosomal flanking genes. Open and shaded arrows represent genomic island ORFs. Shaded blocks indicate homologous ORFs among the *Vibrio *species.

**Figure 3:**
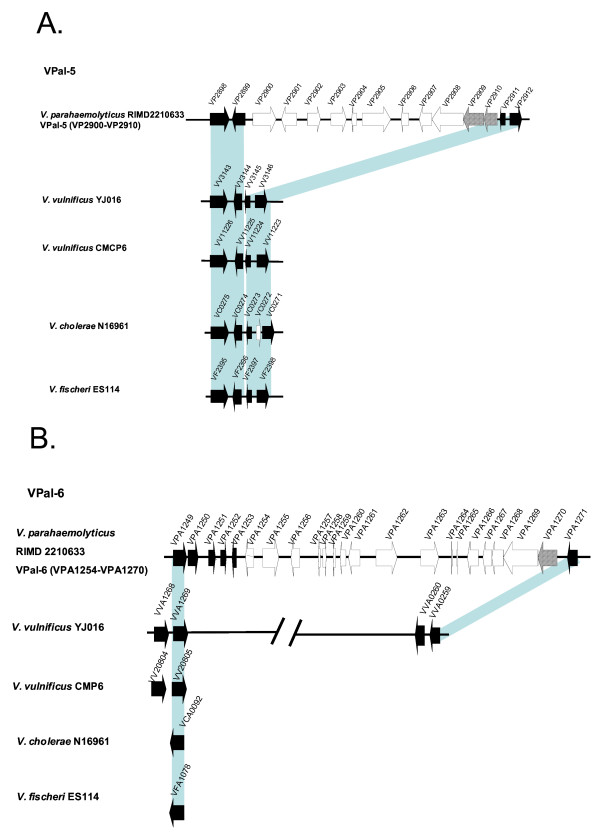
**A and B. Schematic representation of *V. parahaemolyticus *island-5 (VPaI-5) and VPAI-6, and the homologous insertion site among the 4 sequenced *Vibrio *species**. Block arrows represent annotated ORFs. Black filled in ORFs represent core chromosomal flanking genes. Open arrows represent genomic island ORFs. Shaded blocks indicate homologous ORFs among the *Vibrio *species.

Each ORF present in the 7 VPaI regions was analysed systematically by BLAST analysis to determine whether these regions were present in sequenced members of the family *Vibrionaceae*; *V. vulnificus *strains YJ016 and CMCP6, *V. cholerae *N16961, and *V. fischeri *ES114. For all GIs, no homologues were present in these sequenced genomes. However, it was noted that four of the homologous insertion sites tRNA-Met, tmRNA, and two tRNA-Ser loci, did contain unique DNA in each of the four *Vibrio *genome sequences examined. At the tRNA-Met locus (genome location 403769–403844) in the *V. parahaemolyticus *RIMD2210633 sequence, we identified the 24 kb VPaI-1 region (Figure 1A). The VPaI-1 region encompassed ORFs VP0380 to VP0403 and encoded a phage-like integrase, type I restriction endonuclease, haemagglutinin associated protein, transmembrane protein, transcriptional regulators as well as a number of hypothetical proteins. Previously, we identified the homologous tRNA-Met locus as a hotspot for insertion of novel DNA in *V. vulnificus *and *V. cholerae *isolates [22]. At this site in both species a novel genomic island was inserted; in *V. vulnificus *YJ016, a 43 kb *V. vulnificus *island-I (VVI-I) was present and in *V. cholerae *O1 biotype El Tor pandemic strains, the 27 kb *Vibrio *seventh pandemic island-II (VSP-II) was present (Figure 1A) [22,23]. In both *V. vulnificus *CMCP6 and *V. fischeri *ES114 the homologous tRNA-Met insertion site contained no novel DNA. In *V. vulnificus *CMCP6 the two homologous core chromosomal VPaI-1 flanking genes were adjacent to one another, whereas in *V. fischeri *ES114 only one flanking gene was identified (Figure 1A). At the tmRNA site (genome location 674355–675321) in *V. parahaemolyticus *RIMD2210633, the VPaI-2 region is present, which encompassed ORFs VP0635 to VP0643 and encoded an integrase, outer membrane protein, a resolvase, a ribonuclease H1 protein as well as a number of hypothetical proteins (Figure 1B). At the homologous tmRNA site in *V. vulnificus *YJ016 a 20 kb island region was present and in *V. vulnificus *strain CMCP6 a 37 kb region was present [24]. In *V. cholerae*, the 42 kb *Vibrio *Pathogenicity Island-1 (VPI-1) region, which encodes the toxin co-regulated pilus, was present [25]. In *V. fischeri *ES114 at the homologous tmRNA site a 28 kb region was present, which showed homology to prophage genes. At a tRNA-Ser site (genome location 1121082–1121169) in *V. parahaemolyticus *RIMD2210633 the 32 kb VPaI-3 region was present (Figure 2A). The VPaI-3 region encompasses ORFs VP1071 to VP1094 and encoded an integrase, a signal transduction histidine kinase, a helicase, a methyl accepting chemotaxis protein, an AcrBDF family protein as well as numerous hypothetical proteins. In *V. vulnificus *at the homologous insertion site, a 117 kb region was present and in CMCP6 a 29 kb region with homology to the 117 kb region in YJ016 was present (Figure 2A) [24]. At the homologous site in *V. cholerae *the 57 kb VPI-2 region, which encoded neuraminidase and genes required for sialic acid metabolism, was present [26,27]. In *V. fischeri *ES114 at this site no horizontally acquired DNA was present (Figure 2A). In *V. parahaemolyticus *RIMD2210633, at a second tRNA-Ser site (genome location 2239837–2239927) the 17 kb VPaI-4 region was present encompassing ORFs VP2131 to VP2144 (Figure 2B). The VPaI-4 region encoded an integrase, a putative pore forming cytotoxin integrase, an M protein, an ATPase, a histone deacetylase as well as a number of hypothetical proteins. The M protein is a classical bacterial surface expressed virulence factor and the cytotoxin integrase may also be a potential virulence factor. At the homologous tRNA-Ser site in both *V. vulnificus *strains YJ016 and CMCP6 a 7.5 kb region of unique DNA was present (Figure 2B). However, at the homologous sites in *V. cholerae *N16961 and *V. fischeri *ES114 no novel DNA was present, and in *V. cholerae *the core flanking island genes were adjacent to one another (Figure 2B). The 12 kb VPaI-5 region encompassed ORFs VP2900 to VP2910 (Figure 3A). VPaI-5 encoded in addition to two integrases, a number of unknown and hypothetical proteins. The homologous core chromosomal flanking genes of VPaI-5 in *V. vulnificus *strains YJ016 and CMCP6, *V. cholerae *N16961 and *V. fischeri *ES114 were adjacent to one another indicating the absence of novel DNA at this site (Figure 3A). The 27 kb VPaI-6 region encompassed VPA1254 to VPA1270 and encoded an integrase, 3, 4 dihydroxy-2-butanone-4 phos (DHBP) synthase, two putative colicin proteins, a hydrolase and a number of hypothetical proteins. The homologous core chromosomal flanking genes of VPaI-6 in *V. vulnificus *strains YJ016 and CMCP6, *V. cholerae *N16961 and *V. fischeri *ES114 were either dispersed on the genome or were absent (Figure 3B). The 81 kb VPaI-7 region encompassed VPA1312 to VPA1398 encoded a TTS system previously described by Makino and colleagues [19].

### Distribution of VPaI-1 among pre-1996 and post-1995 isolates

Since the tRNA-Met site in *V. cholerae *isolates contained DNA (VSP-II) unique to pandemic isolates, we decided to examine this region further among *V. parahaemolyticus *isolates [23,28]. To determine whether VPaI-1 is unique to pandemic *V. parahaemolyticus*, we examined a collection of 41 *V. parahaemolyticus *strains encompassing various serotypes recovered from a range of geographic regions over a 30 year period. The *V. parahaemolyticus *strains examined included 10 serotype O3:K6 isolates recovered between 1980 and 1995; 17 serotype O3:K6 isolates recovered between 1996 and 1998; 13 isolates representing serotypes O1:KUT, O1:K25 (2 isolates), O2:K28, O3:K4, O3:K53, O4:K8, O4:K11 (2 isolates), O4:K68 (3 isolates) and O8:K22, and 1 strain of undetermined serotype (Table 2). These 41 isolates were further characterized using the differences in *toxRS *nucleotide sequence first described by Matsumoto and co-workers, which they called a group specific PCR (GS-PCR) that has been shown to differentiate post-1995 pandemic strains from non-pandemic and pre-1996 isolates (Table 2) [6]. Of the 41 *V. parahaemolyticus *strains examined using GS-PCR, 25 strains gave a positive PCR band of the expected size and 16 *V. parahaemolyticus *isolates did not yield any PCR product (Table 2). One *toxRS*-positive strain 1364 was previously shown by Wong and colleagues to belong to the old O3:K6 group and in their analysis this strain gave no PCR band with the same primer pair [15]. Our result suggests that the isolate we examined was not the same 1364 strain. We sequenced the housekeeping gene malate dehydrogenase (*mdh*) from strain 1364 using a primer pair designed from the sequenced strain RIMD2210633. The *mdh *nucleotide sequence from strain 1364 differed by only one nucleotide from the RIMD2210633 *mdh *sequence indicating they are very closely related strains. One additional strain gave anomalous results compared to a previous study, where KE10464 was shown by Osawa and colleagues to give positive PCR results with the *toxRS *primer pair [29]. It appears that there may have been a mix up in strain numbering in the distribution of these strains.

**Figure 4:**
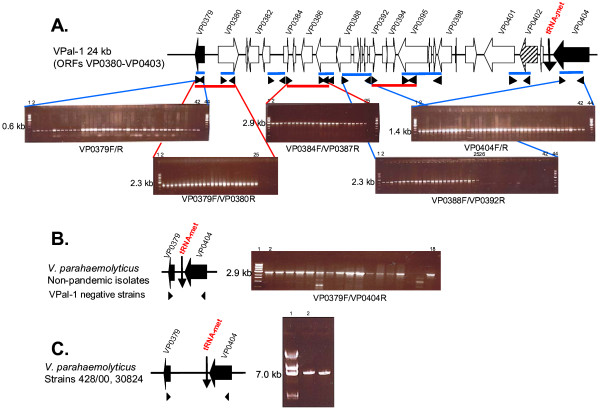
**A. Schematic representation of VPaI-1 showing arrow heads representing primer pairs used in this study**. PCR analysis of the 41 *V. parahaemolyticus *strains is shown representing the results from 3 primer pairs. Lanes 1 and 44 represent size markers; lanes 2 to 42 contain strains analysed in the same order as in Table 2. For overlapping PCR primer pair analysis only VPaI-1-positive strains were examined, lanes 2 to 25 contain strains analysed in the same order as in Table 3. PCR primer pairs used are indicated under each agarose gel. B. Schematic representation of tRNA-Met site in VPaI-1-negative strains. Arrow heads represent primer pair used, which bind to the 5' and 3' core chromosomal flanking genes. PCR analysis of the 17 VPaI-1-negative isolates (lanes 26 to 42) from section A. The band size is marked on the left side of the PCR image. An expected 2.9 kb product was obtained for all strains except 428/00 and 30824 (Lanes 16 and 17). Lane 1 contains marker. C. Schematic representation of tRNA-Met site in VPaI-1 negative strains 428/00 and 30824. Arrow heads represent primer pair used. Long Template Expand PCR on strains 428/00 and 30824 showed 4 kb of DNA at this insertion site. The band size is marked on the left side of the PCR image.

A total of 9 primer pairs were used to determine the distribution of VPaI-1 among 41 *V. parahaemolyticus *natural isolates (Table 3). Six primer pairs were designed within the VPaI-1 region, two primer pairs encompassing each of the VPaI-1 flanking core chromosomal genes, VP0379 and VP0404, and a primer pair comprised of a forward primer from VP0379 and a reverse primer designed from VP0404 (Table 3). Of the 41 *V. parahaemolyticus *strains examined by PCR with 6 primer pairs encompassing the VPaI-1 region (VP0380 to VP0403), 24 strains gave positive PCR bands of the expected sizes indicating the presence of VPaI-1 in these strains (Figure 4A, Table 4). For example, PCR assays with primer pair VP0388F/VP0392R on DNA from the 41 *V. parahaemolyticus *isolates, gave an expected size product of 2.3 kb with 24 strains (Figure 4A, lanes 2 to 25). The 24 PCR positive strains were all recovered post-1995 and had a worldwide distribution (Table 4); 17 *V. parahaemolyticus *serotype O3:K6 strains, 1 serotype O4:K11 strain recovered in Taiwan in 1999, 1 serotype O1:KUT recovered in Bangladesh and 2 serotype O1:K25 strains recovered in Taiwan and Thailand in 1998 and 1999, 3 serotype O4:K68 strains recovered in 1998 and 1999 in Bangladesh, Singapore and Taiwan (Table 4). These VPaI-1 positive isolates were the same set of isolates that gave positive PCR products with the GS-PCR primer pair (Table 2). All *V. parahaemolyticus *strains recovered pre-1996 gave negative PCR results for the six primer pairs encompassing VPaI-1 as well as five strains isolated in Spain and Taiwan between 1998 and 2003 (Table 4). All PCR negative results were confirmed by Southern hybridisation analysis using DNA probes encompassing VP0388 to VP0392. Of the 17 VPaI-1 negative *V. parahaemolyticus *isolates, 16 were negative for the GS-PCR assay, the exception being strain KE10462 (Table 4).

To determine whether in each of the VPaI-1-positve strains the chromosomal insertion site was identical, a primer pair was designed which comprised of a forward primer within VP0379, the 5' core VPaI-1 flanking gene, and a reverse primer within VP0380, the first gene within the island. For all VPaI-1-positive isolates, a 2.3 kb PCR product was obtained (Figure 4A). To confirm that the VPaI-1 regions examined by PCR assays were contiguous within each isolate, overlapping PCR was carried out using three sets of primer pairs on VPaI-1-positive strains only (Table 3). For example, a primer pair encompassing VP0384 to VP0387 was used to amplify a 3 kb product from all VPaI-1-positives isolates, indicating that this region is contiguous with VP0382-VP0384 amongst these isolates (Figure 4A).

**Figure 5:**
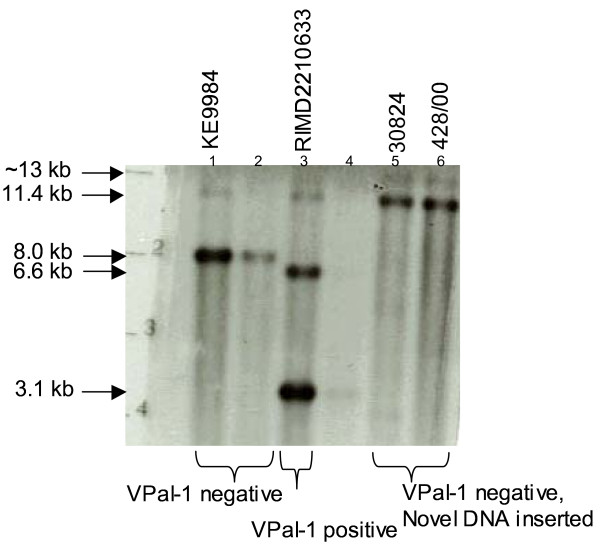
**Southern hybridisation analysis of VPaI-1 negative strains 428/00 and 30824**. PaI-1-negative strains KE9984 and KE9967, VPaI-1-positive strain RIMD2210633 and VPaI-1-negative strains 30824 and 428/00. Genomic DNA was restricted using *Eco*RI and the probe used was constructed from the PCR amplification using primer pair 379F and 404R with strain KE9984 as template. The expected 8 Kb and 11.4 kb band sizes in the VPaI-1 negative strains KE9984 and KE9967 are shown (Lane 1–2). The VPaI-1 positive RIMD2210633 gave the expected 11.4 Kb, 6.6 Kb and 3.1 Kb band sizes (Lane 3). The VPaI-1 negative strains 30824 and 428/00 with novel DNA inserted gave the expected band sizes of 13 Kb and 11.4 Kb.

As expected, positive PCR results with primer pairs marking 5' VP0379 and 3' VP0404 core chromosomal VPaI-1 flanking genes were obtained for all 41 *V. parahaemolyticus *strains, indicating that these genes are present in all isolates (Table 4). Thus, primer pair VP0379F/VP0379R and VP0404F/VP0404R gave a 0.6 kb and 1.4 kb PCR product with all 41 strains tested (Figure 4A). To determine whether in VPaI-1-negative isolates these core chromosomal genes (VP0379 and VP0404) are adjacent to one another, we carried out a PCR assay with a forward primer (379F) designed from VP0379 and a reverse primer (404R) designed from VP0404 (Figure 4B). We found that among the 17 isolates that did not contain the VPaI-1 region, 15 strains gave a positive PCR band of 2.9 kb indicating that in these strains VP0379 and VP0404 are adjacent to one another and the insertion site for VPaI-1 is empty (Table 4, Figure 4B). Two VPaI-1 negative strains, 428/00 and 30824 recovered in Spain 1998 and 1999, did not yield a PCR product with the primer pair 379F and 404R, which suggested that additional DNA may be present in these strains at this region (Figure 4B). We performed long range PCR on both of these isolates and obtained a 7 kb product indicating that in these isolates 4 kb of novel DNA is present (Figure 4C). Further analysis by Southern hybridisation using a DNA probe from the 379F/404R primer pair PCR product from strain KE9984 (VPaI-1 negative strain) showed the expected band sizes from strain 428/00 and 30824. Hybridisation of *Eco*R1 digested RIMD2210633 DNA with the 379/404 probe produced the expected size bands of 11.4 kb, 6.6 kb and 3 kb (Figure 5, lane 3). Hybridisation of *Eco*RI digested KE9984 and KE9967 DNA (VPaI-1 negative strains) with the 379/404 probe gave an expected 11.4 kb and 8 kb bands indicating that in these strains this tRNA-Met site is empty. Strains 30824 and 428/00 gave an approximately 11 kb and 13 kb size bands with the 379/404 DNA probe indicating that in these strains a 4 kb fragment of DNA is present between VP0379 and VP0404 (Figure 5).

### Distribution of VPaI-4 among *V. parahaemolyticus*

To further examine the possible link between the acquisition of novel DNA and the emergence of pandemic isolates, we investigated the presence of three additional regions (VPaI-4, VPaI-5, and VPaI-6) among our collection of 41 *V. parahaemolyticus *isolates. For molecular analyses of VPaI-4, 7 primer pairs designed from strain RIMD2210633 encompassing the 17 kb region and the core 5' and 3' VPaI-4 core chromosomal flanking genes were used (Table 3). The 4 internal VPaI-4 primer pairs gave PCR products of the expected size with DNA template from each of 24 of the 41 isolates examined (Table 5). These were the same 24 isolates that were positive for the presence of VPaI-1 (Table 4). For both the 5' and 3' core chromosomal VPaI-4 flanking primer pairs the expected size PCR products were obtained for all 41 isolates examined indicating that they represent core ancestral genes (Table 5). A PCR assay based on a primer pair binding to chromosomal regions flanking the VPaI-4 region was used to determine whether in VPaI-4-negative strains the flanking genes are adjacent to one another. As expected only the 17 VPaI-4-negative *V. parahaemolyticus *isolates gave a 2.5 kb PCR product with the primer pair designed from the 5' and 3' flanking genes (Table 5). No PCR products were obtained with VPaI-4 positive strains since the distance between the primer binding sites is too large (Table 5). This result showed that in VpaI-4-negative isolates the core chromosomal VPaI-4 flanking genes are adjacent to one another.

**Figure 6:**
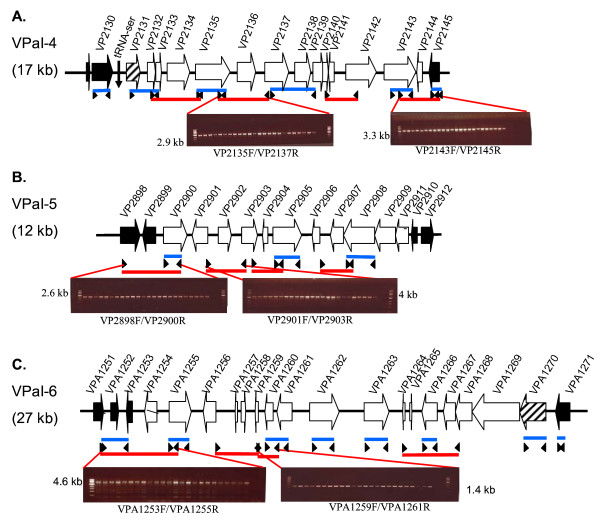
**Schematic representation of VPaI-4, VPaI-5 and VPaI-6 showing arrow heads representing overlapping and insertion site primer pairs used in this study (Table 3)**. Blue bars indicate regions tested for in all strains and red bars indicate regions tested for in VPaI-positive strains only. A. PCR analysis of the 24 VPaI-4-positive strains is shown. Lane 1 represents size marker; lanes 2 to 25 contain strains analysed in the same order as in Table 2. B. PCR analysis of the 24 VPaI-5-positive strains is shown. Lane 1 represents size marker; lanes 2 to 25 contain strains analysed in the same order as in Table 2. C. PCR analysis of the 24 VPaI-6-positive strains is shown. Lane 1 represents size marker; lanes 2 to 25 contain strains analysed in the same order as in Table 2.

To show that in each of the VPaI-4-positive strains that the chromosomal insertion site was identical, a primer pair was designed that comprised of a forward primer within VPaI-4 gene VP2143 and a reverse primer within the 3' core chromosomal flanking gene VP2145 (Table 3). For all VPaI-4-positive isolates, an expected 3.3 kb PCR product was obtained (Figure 6A). To confirm that the VPaI-4 regions examined by PCR assays were contiguous within each isolate, overlapping PCR was carried out using an additional three sets of primer pairs (Table 3). A primer pair encompassing VP2135 to VP2137 was used to amplify a 2.9 kb product from all VPaI-4-positives isolates, indicating that this region is contiguous with VP2137-VP2139 (Figure 6A). Identical results were obtained with primer pairs encompassing VP2132 to VP2135 and VP2139 to VP2142 showing that these regions are contiguous. Thus it appears that in VPaI-4-positive isolates the structure and size of the island among the isolates is similar.

### Distribution of VPaI-5 among *V. parahaemolyticus*

Three primer pairs encompassing the 12 kb VPaI-5 region were used to examine the 41 *V. parahaemolyticus *isolates (Table 3). For all primer pairs, 24 isolates gave a PCR product of the expected size, these were the same 24 isolates that were positive for the presence of VPaI-1 and VPaI-4 (Table 6). The 17 VPaI-5-negative isolates gave a 2.8 kb PCR product with a primer pair designed from the 5' and 3' core chromosomal VPaI-5 flanking genes VP2898 and VP2912 respectively, indicating that these genes are adjacent to one another and no novel DNA is present in these isolates (Table 6).

By PCR assay we show that in each of the VPaI-5-positive strains the insertion site of the island was identical, with a forward primer within the 5' core chromosomal flanking gene VP2898 and a reverse primer within the first gene of VPaI-5 VP2900 (Table 3). From all VPaI-5-positive isolates an expected 4 kb PCR product was obtained indicating that the island is inserted at the same site in all strains tested (Figure 6B). To confirm that the VPaI-5 regions examined by PCR assays were contiguous within each isolate, overlapping PCR was carried out using an additional 3 sets of primer pairs (Table 3). DNA from all VPaI-4-positives isolates by PCR assay amplified a 2.6 kb product with a primer pair encompassing VP2901 to VP2903 (Figure 6B). Identical results were obtained with primer pairs encompassing VP2903 to VP2905 and VP2906 to VP2908 with DNA from VPaI-5-positive isolates as template for PCR assays.

### Distribution of VPaI-6 among *V. parahaemolyticus*

To determine the distribution of VPaI-6 (VPA1254 to VPA1270) encoded on chromosome 2, we used 6 primer pairs spanning the 27 kb region. Similar to the results for VPaI-1, VPaI-4, and VPaI-5, the same set of 24 isolates gave a PCR product of the expected size with each primer pair (Table 7). A primer pair designed within VPA1251 and VPA1253, and a primer pair within VPA1271 all gave a positive PCR product with all strains examined indicating that these genes represent core chromosomal flanking genes (Table 7). The 17 VPaI-6-negative strains gave an expected size PCR product of 0.8 kb with a primer pair designed from the 5' and 3' flanking genes (VPA1253 and VPA1271) indicating that these genes are adjacent to one another in these strains (Table 7).

The insertion site of VPaI-6 among all isolates was examined by PCR assay with a forward primer within the 5' core chromosomal flanking gene VPA1251 and a reverse primer within the first gene of VPaI-6, VPA1255 (Table 3). For DNA from all VPaI-6-positive isolates an expected 4.6 kb PCR product was obtained indicating that the island is inserted at the same site in all strains tested (Figure 6C). To confirm that the VPaI-6 regions examined by PCR assays were contiguous within each isolate, overlapping PCR was carried out using additional sets of primer pairs (Table 3). All VPaI-6-positive isolates by PCR assays, amplified a 4.6 kb PCR product with a primer pair encompassing VPA1251 to VPA1255, a 2 kb PCR product with primer pair VPA1256F/VPA1259R, a 1.4 kb product with a primer pair encompassing VPA1259 to VPA1261, and a 2.9 PCR product with VPA1264F/VPA1268R (Figure 6C). Similar to VPaI-1, VPaI-4 and VPaI-5, it appears that in VPaI-6-positive isolates, the island region has a similar structure and size among all the isolates examined.

### Investigation of excision of VPaI-1, VPaI-4, VPaI-5 and VPaI-6

**Figure 7:**
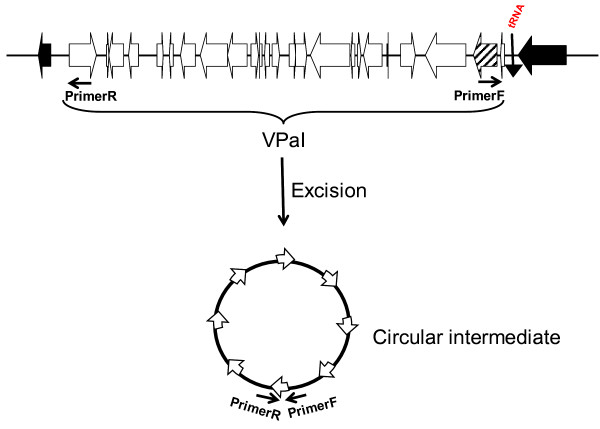
**Schematic representation of chromosomally integrated and extrachromosomal excision product of hypothetical VPaI region**. Location of PCR primers used for analysis of excision products are shown as black arrows labelled PrimerR and PrimerF. Only formation of a circular intermediate or plasmid form of VPaI will allow for the amplification of a PCR product with primer pair primerF and primerR.

The integrase (*int*) genes of VPaI-1 and VPaI-4 exhibit homology to the *int *genes of coliphage P4 and bacteriophage phi CTX, respectively. The *int *genes of VPaI-5 and VPaI-6 show significant identities with other bacteriophages. In addition, VPaI-1, VPaI-4, VPaI-5 and VPaI-6 are flanked by direct repeats, which could be equivalent to the left and right attachment sites (*attL *and *attR*) that result from the integration of phage DNA. Therefore, it is possible that VPaI-1, VPaI-4, VPaI-5 and VPaI-6 may excise from the *V. parahaemolyticus *genome similar to *E. coli *PAIs, which have similar features, and form circular intermediates via site specific recombination between the direct repeats [30]. To determine the stability and potential mobility of VPaI-1, VPaI-4, VPaI-5, and VPaI-6, an inverse PCR assay was carried out to test for the presence of circular intermediates that result from excision of island regions (Figure 7). Total DNA was extracted from overnight cultures treated with and without mitomycin C (induces excision). PCR was carried out using a primer pair oriented towards the 5' and 3' island chromosomal flanking genes (Figure 7). A PCR product can only be amplified if the island regions have excised and formed circular intermediates with the primer binding sites oriented towards one another (Figure 7). Circular intermediates could not be detected under the conditions examined in this study for any of the 4 island regions tested. The deletion rate of these islands maybe very low and our PCR assay may not have been sensitive enough.

## Conclusion

In this study, we identified 7 regions named VPaI-1 to VPaI-7, which had all the features of genomic islands that is aberrant base composition compared to the entire genome, the presence of a phage-like integrase, insertion adjacent to tRNA genes and variable presence among strains. A number of the VPaI regions (VPaI-4 and VPaI-6) encoded putative virulence genes (M protein, hydrolases, cytotoxin integrase, colicins) and therefore these regions may represent potential pathogenicity islands. VPaI-1 encoded a type 1 restriction modification gene cluster, which could potentially be involved in protecting the bacterium from viral infections (Virus resistance island). However, all the VPaI regions encoded a large proportion of hypothetical and unknown proteins. We examined the 24 kb VPaI-1 region among a range of pandemic and non-pandemic *V. parahaemolyticus *isolates recovered over the past 33 years. We determined that VPaI-1 was unique to the pandemic group of *V. parahaemolyticus *strains isolated after 1995. Further analysis of 3 additional regions, VPaI-4, VPaI-5, and VPaI-6 showed that these too were unique to the pandemic clone, indicating that the acquisition of novel DNA by horizontal gene transfer has played an important role in the emergence of these strains. The 12 kb VPaI-5 region encompassing VP2900 to VP2910 encodes ORF VP2905, which was previously shown to be associated with the pandemic group of *V. parahaemolyticus *isolates [31]. The VPaI-5 region was previously noted by Okura and co-workers to encode a phage-like protein and may therefore encode a phage [14]. Previous studies examining the presence of strain specific DNA among pathogenic *V. cholerae *isolates have shown that the acquisition of DNA encoding virulence genes has played a crucial role in the emergence of pandemic isolates of this species [22,25,27,28,32]. The emergence of a novel epidemic *V. cholerae *O139 serogroup in 1992 was shown to have resulted from the horizontal transfer of the O139 gene cluster into an O1 serogroup strain as well as the acquisition of a capsule polysaccharide and an integrative conjugative element [33-37]. The insertion of 4 of our VPaI regions adjacent to tRNA genes has previously been described for a number of GIs in Gram-negative bacteria [38-40]. More recently it has been noted that there is a bias in the integration of GIs into specific tRNA loci [41,42]. As the VPaI regions described in this study are examined further their role in the emergence of a pandemic clone will be elucidated. The recent discovery that *V. cholerae *isolates are naturally competent in the presence of chitin, an abundant substrate in the aquatic environment, may indicate a possible mechanism of DNA acquisition among the *Vibrionaceae *[43].

## Methods

### Genome sequences

Complete nucleotide sequence and annotation was retrieved and downloaded from NCBI ftp site for *V. parahaemolyticus *RIMD2210633 (NC_004603, NC_004605) [19,44].

### Identification of genomic islands (GIs)

Several criteria were used in this study to identify GIs within the sequenced strain RIMD2210633. Firstly, the complete genome sequence of RIMD2210633 was examined for regions of sequence composition bias such as aberrant G+C percentage compared to the entire chromosome. Regions of greater than 10 kb with a G+C content that differed from the host genome were then examined for the presence of integrase and transposase genes. The regions encoding a prophage (VP1549 to VP1589) and a superintegron (VP1765 to VP1866) were not examined further. Seven candidate GI regions were analysed for compositional bias of dinucleotide frequency. The dinucleotide frequency analysis calculates the genomic dissimilarity values δ* (the average dinucleotide relative abundance difference) between GI sequences and the *V. parahaemolyticus *RIMD2210633 genome sequence using a web based application, deltarho-web [20,21,45].

The dinucleotide frequency analysis shows that each genome has a typical dinucleotide frequency (called the genome signature ρ*) and that related species have a similar genome signature [46,47]. Van Passel's method also calculates the plot position (in %) of the GI sequence in the δ*, compared to fragment number plot of the complete genome divided in non-overlapping fragments of equal size as the GI sequence, since the length of the GI sequence is important in calculating the relevance of the value of δ* [20,48]. Putative GI regions that fulfilled the criteria of aberrant G+C and dinucleotide frequency were then examined for the presence of insertion elements, flanking direct repeats, and proximity of tRNA. The 7 GI regions identified in *V. parahaemolyticus *strain RIMD2210633 by the above criteria were further analysed for sequence similarities using the BLAST algorithm to determine whether the ORFs present in each island are found among the other sequenced *Vibrio *genomes, *V. cholerae *N16961, *V. vulnificus *YJ016 and CMCP6, and *V. fischeri *ES114.

### Bacterial strains

A total of 41 *V. parahaemolyticus *isolates were used in this study (Table 2). The source of the 41 *V. parahaemolyticus *isolates was temporally (1970 to 2003) and geographically (Asia, Europe and South America) widespread and encompassed 10 different serotypes (Table 2). *V. parahaemolyticus *strains were grown in Luria-Bertani (LB) broth containing 3% NaCl. Stock cultures were stored at -80°C in LB broth containing 30% glycerol.

### Molecular analysis

Of the 7 regions identified by bioinformatic analyses, we examined 4 regions in detail to determine their distribution among a collection of *V. parahaemolyticus *natural isolates. For polymerase chain reaction (PCR) assays, total DNA was extracted from overnight cultures in 3% NaCl LB broth using G-nome DNA isolation kit (Bio101, USA). To determine the presence of VPaI-1, for example, PCR assays using 6 primer pairs designed from ORFs VP0380 to VP0403 of the sequenced RIMD2210633 genome were used (Table 3, Figure 4A). In addition, primer pairs VP379F/VP379R and VP404F/VP404R were designed to amplify the gene VP0379, which is immediately 5' of VP0380 and the gene VP0404, which is immediately 3' of the tRNA-Met gene, the insertion site of VPaI-1. To examine isolates that did not contain the VPaI-1 region, a single primer pair (379F/404R) consisting of a forward primer 379F and a reverse primer 404R was also used in this study to determine whether VP0379 and VP0404 are directly adjacent to one another in these VPaI-1 negative strains (Table 3, Figure 4B). The 41 *V. parahaemolyticus *strains were also examined with the GS-PCR primer pair that specifically amplifies a 651 bp PCR product from new O3:K6 and related pandemic isolates [6,12-14]. All PCR reactions were performed in volumes of 25 μl containing 40 ng DNA, 10 pmol primer and 1 U Taq polymerase with amplification conditions of 96°C for 3 min, followed by 30 cycles of 94°C for 30 sec, 45–60°C (depending on primer pair) for 30 sec and 72°C for 1–4 min (depending on expected PCR product size) and 72°C for 10 minutes. The sequenced *V. parahaemolyticus *strain RIMD2210633, which contains the VPaI-1 region was used as a positive PCR control. Long range PCR reactions were performed with Elongase ^® ^Enzyme Mix (Invitrogen, Carlsbad, CA, USA). The thermal profile for the long-range PCR amplification included an initial pre-amplification denaturation cycle of 94°C for 30 s, followed by 33 cycles of 94°C for 30 s, 64°C for 30 s and 68°C for 20 minutes. The primer pairs used to investigate the presence of VPaI-4, VPaI-5 and VPaI-6 among the collection of *V. parahaemolyticus *isolates are listed in Table 3. For all island-positive strains, overlapping PCR analysis was carried out with 3 to 4 additional PCR primer pairs per island to confirm that each island region was located at the same insertion site and that the structure of each island is similar amongst all the strains examined (Table 3). Southern hybridisation analysis was performed on all VPaI-1 negative strains to confirm PCR negative results using a probe generated from the PCR product derived from primer pair VP0388F/VP0392 using reference strain RIMD2210633 as template. DNA from each VPaI-1 negative strain was digested with the restriction enzyme *Eco*RI (Roche Molecular Biochemicals) and the fragments were separated by electrophoresis in 0.6% TAE agarose. DNA fragments were transferred to nylon membrane by a posiblotter (Stratagene). Probe DNAs were labelled using the ECL direct nucleic acid labelling system (Amersham Pharmacia Biotech) and positive hybridization was detected by the ECL chemiluminescent substrate. Southern analyses were also preformed on VPaI-4, VPaI-5 and VPaI-6 negative strains using a probe generated from each of these island regions in RIMD2210633. Nucleotide sequence analysis of the *mdh *locus was preformed using a primer pair designed from VP0325.

### Detection of excision of VPaI-1, VPaI-4, VPaI-5 and VPaI-6

An inverse PCR assay was carried out to test for the presence of circular intermediates that result from excision of island regions. Five VPaI-positive and one VPaI-negative *V. parahemolyticus *strains were cultured in LB 3% NaCl broth. The broth was inoculated from single colonies taken from overnight plate cultures and incubated overnight on an orbital shaker at 30°C. The culture was induced to the lytic cycle by the addition of 40 ngml^-1 ^mitomycin C (Sigma-Aldrich). Induced cultures were further incubated for 8–10 h on an orbital shaker at 30°C. Cultures were centrifuged at 5000 g for 10 min to pellet bacterial cells and the supernatant fluids were filtered through 0·45μm membranes (Millipore). Aliquots of 2 μl were subsequently used as template for inverse PCR. Primer pairs oriented towards the 5' and 3' island chromosomal flanking genes were used to screen the filtrate (Table 3). A PCR product can only be amplified if the island regions have excised and formed circular intermediates with the primer binding sites oriented towards one another.

## Abbreviations

TDH Thermostable Direct Hemolysin

GI Genomic Islands

TTS Type III Secretion

VPaI *V. parahaemolyticus *Island

tRNA Transfer RNA

ORFs Open Reading Frames

VVI-I *V. vulnificus *Island-I

GF Genomic Fragments

PAI Pathogenicity Island

PCR Polymerase Chain Reaction

GS-PCR Group Specific PCR

## Authors' contributions

CH performed bioinformatic and molecular analyses, AMQ performed long range PCR, FJR performed inverse PCR, and AMQ and FJR assisted in primer design and genome sequence analyses. EFB planned the research, EFB and CH wrote the manuscript and AMQ and FJR helped with preparation and review of the manuscript. All authors read and approved the final manuscript.

## Figures and Tables

**Table 1:** Genomic Islands (Gis) identified in *V. parahaemolyticus *RIMD2210633 genome sequence.

**GIs**	**Location (bp)(ORFs)**	**Directrepeat size**	**SizeKb**	**Int/Tnp**	**tRNAsite**	**%GC**	1000X 6*	% GF withlower 6*
VPaI-1	381054–403433 (VP0380–VP0403)	45	24	Int	tRNA-Met	42	69.1	97.8
VPaI-2	6660707–674355 (VP0635–VP0643)	10	10	Int	tmRNA	45	76.7	93.9
VPaI-3	1121252–1152668 (VP1071–VP1094)	-	32	Int	tRNA-Ser	42	53.1	98.8
VPaI-4	2240007–2256166 (VP2131–VP2144)	30	17	Int	tRNA-Ser	39	89.3	99.5
VPaI-5	3084846–3099979 (VP2900–VP2910)	13	12	Int	-	38	130.5	100
VPaI-6	1325821–1352643 (VPA1253–VPA1270)	8	27	Int	-	43	72.1	97.2
VPaI-7	1390967–1501509 (VPA1312–VPA1398)	8	81	Tnp	-	39	77.4	100

δ*-average dinucleotide relative abundance difference

Int/Tnp- Integrase/Transposase

GF-Genome Fragments

**Table 2:** Bacterial strains used in this study.

**Strain**	**Year Isolated**	**Serovar**	**Country of Isolation**	**Source**	**GS-PCR**	***tdh***	**Reference**
RIMD2210633	1996	O3:K6	Japan	Human	+	+	[16]
97 LPV 2	1997	O3:K6	Laos	Human	+	+	[7]
VP2	1998	O3:K6	Korea	Human	+	+	[7]
VP47	1998	O3:K6	Thailand	Human	+	+	[7]
VP208	1997	O3:K6	India	Human	+	+	[15]
VP81	1996	O3:K6	India	Human	+	+	[7]
NIIDK7	1998	O3:K6	Japan	Human	+	+	[29]
KE10457	1998	O3:K6	Japan	Human	+	+	[29]
KE10472	1998	O3:K6	Japan	Human	+	+	[29]
KE10484	1998	O3:K6	Japan	Human	+	+	[29]
KE10495	1996	O3:K6	Japan	Human	+	+	[29]
KE10527	1998	O3:K6	Japan	Food	+	+	[29]
MW2	1998	O3:K6	Peru	Human	+	+	This study
MW3	1998	O3:K6	Peru	Human	+	+	This study
MW4	1998	O3:K6	Peru	Human	+	+	This study
MW5	1998	O3:K6	Peru	Human	+	+	This study
MW6	1998	O3:K6	Peru	Human	+	+	This study
A6	1999	O1:K25	Thailand	Human	+	+	[15]
1362	1999	O4:K68	Taiwan	Human	+	+	[15]
AN- 5034	1998	O4:K68	Bangladesh	Human	+	+	[7]
A18	1998	O4:K68	Singapore	Human	+	+	[15]
1364	1999	O4:K11	Taiwan	Human	+	+	[15]
AN-16000	1998	O1:KUT	Bangladesh	Human	+	+	[7]
1347	1998	O1:K25	Taiwan	Human	+	+	[15]

UCM V586	2003	O8:K22	Spain	Mollusc	-	-	[7]
KE10443	1995	O3:K6	Japan	Human	-	-	[29]
KE10464	1988	O3:K6	Japan	Food	-	-	[29]
AQ4235	1987	O3:K6	Thailand	Human	-	-	[15]
AQ4299	1987	O3:K6	Thailand	Human	-	-	[15]
KE10462	1986	O3:K6	Japan	Food	+	-	[29]
AQ4037	1985	O3:K6	Maldives	Human	-	-	[15]
KE10461	1982	O3:K6	Japan	Environmental	-	-	[29]
KE9967	1981	O3:K6	Japan	Human	-	+	[29]
KE9984	1981	O3:K6	Japan	Human	-	-	[29]
U-5474	1980	O3:K6	Bangladesh	Human	-	+	[15]
ATCC43996	1970	O3:K4	U.K	Human	-	+	[7]
UCM V553	2003	O3:K53	Spain	Mollusc	-	-	[7]
UCM V493	2002	O2:K28	Spain	Sediment	-	-	[7]
30824	1999	O4:K11	Spain	Human	-	+	[7]
428/00	1998	N/A	Spain	Human	-	+	[7]
1324	1998	O4:K8	Taiwan	Human	-	+	[15]

**Table 3:** Primer Pairs used in this study.

**Primers**	**Anneal Temp**	**Product size**	**Primer sequences**
**VPaI-1**			
VP0379F-VP0379R	52.2	589nt	Forward 5'-CAGTAATCAAAGTCGGAGC -3' Reverse 5'-ATAACTGCGAGAGGTGC -3'
VP0380F-VP0380R	50.7	1004nt	Forward 5'-ATCTCTGGGATCAGTCAGG -3' Reverse 5'-GCGATAACGAGTCACTG -3'
VP0382F-VP0384R	50.4	1809nt	Forward 5'-CGTATTCCATGAATGTCGAG -3' Reverse 5'-GCTCTGCGTAATGCTTC -3'
VP0387F-VP0387R	51.9	1422nt	Forward 5'-CCTAGATTTCAAAGGAAGTGCG-3' Reverse 5'-GTCACGGATATGTTGTCTTG-3'
VP0388F-VP0392R	53.4	2256nt	Forward 5'-GTTGGTCTTAATGCCCG -3' Reverse 5'-ACGTACTGTTCAAGCCGAC -3'
VP0395F-VP0398R	53.2	2393nt	Forward 5'-TGTCCGCCAAAAACAGG -3' Reverse 5'-GCTGAGATCATCGAAAGTTACG -3'
VP0401F-VP0402R	51	1171nt	Forward 5'-GTCTTCTGTGAATGACTGC -3' Reverse 5'-TTAGCTTCTTCGTCACCCC -3'
VP0404F-VP0404R	52.1	1397nt	Forward 5'-CATGGGTATCAAGGTAGTAG -3' Reverse 5'-TCTCCATTGAGATTGGC -3'
379F-404R	55.1	2842nt	Forward 5'-ACCATAACTGCGAGAGGTGC -3' Reverse 5'-TGGTCGTACAACAGACCCAG -3'
**Overlapping PCR Primers**			
VP0379R-VP0380R	50	2304nt	Forward 5'-ATAACTGCGAGAGGTGC -3' Reverse 5'-GCGATAACGAGTCACTG -3'
VP0384R-VP0387R	51	2990nt	Forward 5'-CCGTAAGAGAAGAACGTG-3' Reverse 5'-CGAGTTAATGCAAAACTGGG-3'
VP0384R-VP0387R	53	2035nt	Forward 5'-ATTGGGGTTGGATGTCGTGG-3' Reverse 5'-CGAACGTGTTCAATACTACG-3'
**VPaI-4**			
VP2130F-VP2130R	52.3	1575nt	Forward 5'-GCTTCGATGTTGATAGGC -3' Reverse 5'-GATGAGAGCAAGTTCTCG -3'
VP2131F-VP2133R	51.3	1785nt	Forward 5'-CACGTATTTCCACTCCAG -3' Reverse 5'-CCCATACTTCACCATCTTTC -3'
VP2135F-VP2135R	50.4	1653nt	Forward 5'-GGTAAGAGTGAAGAGGTTCAG-3' Reverse 5'-CCGACTGTAAATCTCATCG-3'
VP2137F-VP2139R	50.9	2914nt	Forward 5'-TCGTGTGAAATGCGAAC -3' Reverse 5'-CGACGTAGACTTTTAGCC -3'
VP2143F-VP2143R	50.5	1354nt	Forward 5'-GCAGTCGAACTTGAAGAC -3' Reverse 5'-ATGACCTCTCTGCATCC -3'
VP2145F-VP2145R	52	727nt	Forward 3'-TATCCACCACCCACAAC-3' Reverse 3'-ATGTGCAAGCTGAGTGG-3'
2130F- 2145R	53.2	2851nt	Forward 5'-GCTTCGATGTTGATAGGC -3' Reverse 5'-ATGTGCAAGCTGAGTGGGTG -3'
**Overlapping PCR primers**			
VP2143F-VP2145R	49	3291nt	Forward 5'-GCAGTCGAACTTGAAGAC -3' Reverse 3'-ATGTGCAAGCTGAGTGG-3'
VP2132F-VP2135R	50	2387nt	Forward 5'-TCCCTCGTTTCTTGCTC-3' Reverse5'-CTTGTCACAATGATCTCCTG-3'
VP2135F-VP2137R	51	2900nt	Forward 5'-GCTGGATTAGGACTTGATAC-3' Reverse5'-TACTAACCTCCCATCGG-3'
VP2140F-VP2142R	52	2562nt	Forward 5'-GGCGTAATTTCTCAGATTCC-3' Reverse 5'-GCTAAACGTGTTGCTGTTG-3'
**VPaI-5**			
VP2900F-VP2900R	50.3	1377nt	Forward 5'-GGGGAACCAGTTATAGG-3' Reverse 5'-ACCGTGAGACGCAGTATCAG -3'
VP2905F-VP2905R	50.7	2035nt	Forward 5'-CTGATGAGCAGTTTCCG -3' Reverse 5'-TCCATTGTTGTTGCAGG -3'
VP2908F-VP2908R	50.6	2313nt	Forward 5'-GGCATGAAATCTTGCAG-3' Reverse 5'-CTCACATAGTTACATTGCGG
2898F-2912R	59.3	2800nt	Forward 5'-CAGCCAAGACCACAAAC-3' Reverse 5'-AATCCCTAGCAGGTTGC-3'
**Overlapping PCR primers**			
VP2898F-VP2900R	52	3988nt	Forward 5'-CAGCCAAGACCACAAAC-3' Reverse 5'-ACCGTGAGACGCAGTATCAG -3'
VP2901F-VP2903R	52	2577nt	Forward 5'-AACGCCAAAGTTGCCTG -3' Reverse 5'-CCGTAAGTAAAGGTTCATCTGC -3'
VP2903F-VP2905R	53	2330nt	Forward 5'-TCACGTTTGAAGCTCGC -3' Reverse 5'-GTGCCGTATTCTCTTTGTAG -3'
VP2906F-VP2908R	51.2	2197nt	Forward 5'-GCCCATACAAGTATTCACTC -3' Reverse 5'-TAACCCACAACGATCCG -3'
**VPaI-6**			
VPA1251F-VPA1253R	51.3	1169nt	Forward 5'-TCGTAAACACTGCCAGC -3' Reverse 5'-CTAGCAACGGTGATAGC -3'
VPA1255F-VPA1255R	51.1	2098nt	Forward 5'-GATGGTTGTGATGAGTTTGC -3' Reverse 5'-TGATGAAGCGATATGCC -3'
VPA1260F-VPA1261R	51.5	2033nt	Forward 5'-GGCTTTAGGTACTGCTACC -3' Reverse 5'-GGAATCACGTTCTCTGG -3'
VPA1262F-VPA1262R	54.1	1851nt	Forward 5'-GTGACTTCGAGTTCATTTGC -3' Reverse 5'-GCAACAGTGCATCAATTTGCTC -3'
VPA1263F-VPA1263R	53	2144nt	Forward 5'-CTACGAAGGTAATCTCGC -3' Reverse 5'-TACCCGCATCAGCTAAC -3'
VPA1266F-VPA1266R	52.5	1879nt	Forward 5'-ATCCCTTATCGCTCGAC-3' Reverse 5'-CGGATTGTGTTATCTCG-3'
VPA1270F-VPA1270R	53.2	2225nt	Forward 5'-TGGAACACGCATCCTAC-3' Reverse 5'-ACGACTGAGTTGCATCGAG-3'
VPA1271F-VPA1271R	51.9	432nt	Forward 5'-CAGCACACTTTTACTGGC -3' Reverse 5'-TGCATCGAACTAACCGC -3'
1253F-1271R	53	782nt	Forward 5'-ACTTCTGGCTGCGTTTC -3' Reverse 5'-CACTTTTACTGGCTTTCAGC -3'
**Overlapping PCR primers**			
VPA1251F-VPA1255R	48	4590nt	Forward 5'-TCGTAAACACTGCCAGC -3' Reverse 5'-TGATGAAGCGATATGCC -3'
VPA1256F-VPA1259R	50.7	1995nt	Forward 5'-GCCGAGTCAATAAATGTTGCAC -3' Reverse 5'-TTCTAGTTGAACGACGC -3'
VPA1259F-VPA1261R	52.2	1367nt	Forward 5'-AACGTCAATTTCTGGGG -3' Reverse 5'-GCATCTGAGACAAAGACTAAGG -3'
VPA1264F-VPA1268R	53	2885nt	Forward 5'-CGAACCTTAAATGGACG -3' Reverse 5'-GACTAATGGCAAGTGGG -3'
**Inverse PCR primers**			
VP0401F-VP0380R	49	4596nt	Forward 5'-GTCTTCTGTGAATGACTGC -3' Reverse 5'-GCGATAACGAGTCACTG -3'
VP2143F-VP2133R	51	4614nt	Forward 5'-GCAGTCGAACTTGAAGAC -3' Reverse 5'-CCCATACTTCACCATCTTTC -3'
VP2910F- VP2900R	56	2939nt	Forward 5'-CAACATGTACCCAGAGTACG -3' Reverse 5'-ACCGTGAGACGCAGTATCAG -3'
VPA1270F-VPA1255R	48	4462nt	Forward 5'-TGGAACACGCATCCTAC-3' Reverse 5'-GCTGGTGACCTCGATAC-3'
**Genotypic primer pairs**			
GS-VP.1 F-GS-VP.2 R	45	651nt	Forward 5'-TAATGAGGTAGAAACA-3' Reverse 5'-ACGTAACGGGCCTACA-3'
VP0325F-VP0325R	54	901nt	Forward 5'-TGAAAGTAGCCGTTATTGG -3' Reverse 5'-CCATTTAGCGTTTCTAGCATTC-3'

**Table 4:** Occurrence and distribution of Group Specific and VPaI-1 genesamong *V. parahaemolyticus *isolates.

**Strains**	**Serovar**	**GS-PCR**	**VP 0379**	**VP 0380**	**VP 0382–84**	**VP 0387**	**VP 0388–92**	**VP 0395–98**	**VP 0401–02**	**VP 0404**	**379F-404R**
RIMD 2210633	O3:K6	+	+	+	+	+	+	+	+	+	-
97 LPV 2	O3:K6	+	+	+	+	+	+	+	+	+	-
VP2	O3:K6	+	+	+	+	+	+	+	+	+	-
VP47	O3:K6	+	+	+	+	+	+	+	+	+	-
VP208	O3:K6	+	+	+	+	+	+	+	+	+	-
VP81	O3:K6	+	+	+	+	+	+	+	+	+	-
NIIDK7	O3:K6	+	+	+	+	+	+	+	+	+	-
KE10457	O3:K6	+	+	+	+	+	+	+	+	+	-
KE10472	O3:K6	+	+	+	+	+	+	+	+	+	-
KE10484	O3:K6	+	+	+	+	+	+	+	+	+	-
KE10495	O3:K6	+	+	+	+	+	+	+	+	+	-
KE10527	O3:K6	+	+	+	+	+	+	+	+	+	-
MW2	O3:K6	+	+	+	+	+	+	+	+	+	-
MW3	O3:K6	+	+	+	+	+	+	+	+	+	-
MW4	O3:K6	+	+	+	+	+	+	+	+	+	-
MW5	O3:K6	+	+	+	+	+	+	+	+	+	-
MW6	O3:K6	+	+	+	+	+	+	+	+	+	-
A6	O1:K25	+	+	+	+	+	+	+	+	+	-
1362	O4:K68	+	+	+	+	+	+	+	+	+	-
AN-5034	O4:K68	+	+	+	+	+	+	+	+	+	-
A18	O4:K68	+	+	+	+	+	+	+	+	+	-
1364	O4:K11	+	+	+	+	+	+	+	+	+	-
AN-16000	O1:KUT	+	+	+	+	+	+	+	+	+	-
1347	O1:K25	+	+	+	+	+	+	+	+	+	-

UCM V586	O8:K22	-	+	-	-	-	-	-	-	+	+
KE10443	O3:K6	-	+	-	-	-	-	-	-	+	+
KE10464	O3:K6	-	+	-	-	-	-	-	-	+	+
AQ4235	O3:K6	-	+	-	-	-	-	-	-	+	+
AQ4299	O3:K6	-	+	-	-	-	-	-	-	+	+
KE10462	O3:K6	+	+	-	-	-	-	-	-	+	+
AQ4037	O3:K6	-	+	-	-	-	-	-	-	+	+
KE10461	O3:K6	-	+	-	-	-	-	-	-	+	+
KE9967	O3:K6	-	+	-	-	-	-	-	-	+	+
KE9984	O3:K6	-	+	-	-	-	-	-	-	+	+
U-5474	O3:K6	-	+	-	-	-	-	-	-	+	+
ATCC43996	O3:K4	-	+	-	-	-	-	-	-	+	+
UCM V553	O3:K53	-	+	-	-	-	-	-	-	+	+
UCM V495	O2:K28	-	+	-	-	-	-	-	-	+	+
30824	O4:K11	-	+	-	-	-	-	-	-	+	-
428/00	N/A	-	+	-	-	-	-	-	-	+	-
1324	O4:K8	-	+	-	-	-	-	-	-	+	+

**Table 5:** Distribution of VPaI-4 region (ORFs VP2131 to VP2144) among *V. parahaemolyticus *natural isolates.

**Strain**	**Serovar**	**VP2130**	**VP2131–33**	**VP2135**	**VP2137–39**	**VP2143**	**VP2145**	**2130–2145**
RIMD 2210633	O3:K6	+	+	+	+	+	+	-
97 LPV 2	O3:K6	+	+	+	+	+	+	-
VP2	O3:K6	+	+	+	+	+	+	-
VP47	O3:K6	+	+	+	+	+	+	-
VP208	O3:K6	+	+	+	+	+	+	-
VP81	O3:K6	+	+	+	+	+	+	-
NIIDK7	O3:K6	+	+	+	+	+	+	-
KE10457	O3:K6	+	+	+	+	+	+	-
KE10472	O3:K6	+	+	+	+	+	+	-
KE10484	O3:K6	+	+	+	+	+	+	-
KE10495	O3:K6	+	+	+	+	+	+	-
KE10527	O3:K6	+	+	+	+	+	+	-
MW2	O3:K6	+	+	+	+	+	+	-
MW3	O3:K6	+	+	+	+	+	+	-
MW4	O3:K6	+	+	+	+	+	+	-
MW5	O3:K6	+	+	+	+	+	+	-
MW6	O3:K6	+	+	+	+	+	+	-
A6	O1:K25	+	+	+	+	+	+	-
1362	O4:K68	+	+	+	+	+	+	-
AN-5034	O4:K68	+	+	+	+	+	+	-
A18	O4:K68	+	+	+	+	+	+	-
1364	O4:K11	+	+	+	+	+	+	-
AN-16000	O1:KUT	+	+	+	+	+	+	-
1347	O1:K25	+	+	+	+	+	+	-

UCM V586	O8:K22	+	-	-	-	-	+	+
KE10443	O3:K6	+	-	-	-	-	+	+
KE10464	O3:K6	+	-	-	-	-	+	+
AQ4235	O3:K6	+	-	-	-	-	+	+
AQ4299	O3:K6	+	-	-	-	-	+	+
KE10462	O3:K6	+	-	-	-	-	+	-
AQ4037	O3:K6	+	-	-	-	-	+	+
KE10461	O3:K6	+	-	-	-	-	+	+
KE9967	O3:K6	+	-	-	-	-	+	+
KE9984	O3:K6	+	-	-	-	-	+	+
U-5474	O3:K6	+	-	-	-	-	+	+
ATCC43996	O3:K4	+	-	-	-	-	+	+
UCM V553	O3:K53	+	-	-	-	-	+	+
UCM V493	O2:K28	+	-	-	-	-	+	+
30824	O4:K11	+	-	-	-	-	+	+
428/00	N/A	+	-	-	-	-	+	+
1324	O4:K8	+	-	-	-	-	+	+

**Table 6:** Distribution of VPaI-5 region (ORFs VP2900 to VP2910) among *V. parahaemolyticus *natural isolates.

**Strain**	**Serovar**	**VP 2901**	**VP 2905**	**VP 2908**	**2898–2912**
RIMD 2210633	O3:K6	+	+	+	-
97 LPV 2	O3:K6	+	+	+	-
VP2	O3:K6	+	+	+	-
VP47	O3:K6	+	+	+	-
VP208	O3:K6	+	+	+	-
VP81	O3:K6	+	+	+	-
NIIDK7	O3:K6	+	+	+	-
KE10457	O3:K6	+	+	+	-
KE10472	O3:K6	+	+	+	-
KE10484	O3:K6	+	+	+	-
KE10495	O3:K6	+	+	+	-
KE10527	O3:K6	+	+	+	-
MW2	O3:K6	+	+	+	-
MW3	O3:K6	+	+	+	-
MW4	O3:K6	+	+	+	-
MW5	O3:K6	+	+	+	-
MW6	O3:K6	+	+	+	-
A6	O1:K25	+	+	+	-
1362	O4:K68	+	+	+	-
AN-5034	O4:K68	+	+	+	-
A18	O4:K68	+	+	+	-
1364	O4:K11	+	+	+	-
AN-16000	O1:KUT	+	+	+	-
1347	O1:K25	+	+	+	-

UCM V586	O8:K22	-	-	-	+
KE10443	O3:K6	-	-	-	+
KE10464	O3:K6	-	-	-	+
AQ4235	O3:K6	-	-	-	+
AQ4299	O3:K6	-	-	-	+
KE10462	O3:K6	-	-	-	+
AQ4037	O3:K6	-	-	-	+
KE10461	O3:K6	-	-	-	+
KE9967	O3:K6	-	-	-	+
KE9984	O3:K6	-	-	-	+
U-5474	O3:K6	-	-	-	+
ATCC43996	O3:K4	-	-	-	+
UCM V553	O3:K53	-	-	-	+
UCM V493	O2:K28	-	-	-	+
30824	O4:K11	-	-	-	+
428/00	N/A	-	-	-	+
1324	O4:K8	-	-	-	+

**Table 7:** Distribution of VPaI-6 region (ORFs VPA1254 to VPA1270) among *V. parahaemolyticus *natural isolates.

**Strain**	**Serovar**	**VPA1251–53**	**VPA1255**	**VPA1260**	**VPA1262**	**VPA1263**	**VPA1266**	**VPA1270**	**VPA1271**	**1253–1271**
RIMD 2210633	O3:K6	+	+	+	+	+	+	+	+	-
97 LPV 2	O3:K6	+	+	+	+	+	+	+	+	-
VP2	O3:K6	+	+	+	+	+	+	+	+	-
VP47	O3:K6	+	+	+	+	+	+	+	+	-
VP208	O3:K6	+	+	+	+	+	+	+	+	-
VP81	O3:K6	+	+	+	+	+	+	+	+	-
NIIDK7	O3:K6	+	+	+	+	+	+	+	+	-
KE10457	O3:K6	+	+	+	+	+	+	+	+	-
KE10472	O3:K6	+	+	+	+	+	+	+	+	-
KE10484	O3:K6	+	+	+	+	+	+	+	+	-
KE10495	O3:K6	+	+	+	+	+	+	+	+	-
KE10527	O3:K6	+	+	+	+	+	+	+	+	-
MW2	O3:K6	+	+	+	+	+	+	+	+	-
MW3	O3:K6	+	+	+	+	+	+	+	+	-
MW4	O3:K6	+	+	+	+	+	+	+	+	-
MW5	O3:K6	+	+	+	+	+	+	+	+	-
MW6	O3:K6	+	+	+	+	+	+	+	+	-
A6	O1:K25	+	+	+	+	+	+	+	+	-
1362	O4:K68	+	+	+	+	+	+	+	+	-
AN-5034	O4:K68	+	+	+	+	+	+	+	+	-
A18	O4:K68	+	+	+	+	+	+	+	+	-
1364	O4:K11	+	+	+	+	+	+	+	+	-
AN-16000	O1:KUT	+	+	+	+	+	+	+	+	-
1347	O1:K25	+	+	+	+	+	+	+	+	-

UCM V586	O8:K22	+	-	-	-	-	-	-	+	+
KE10443	O3:K6	+	-	-	-	-	-	-	+	+
KE10464	O3:K6	+	-	-	-	-	-	-	+	+
AQ4235	O3:K6	+	-	-	-	-	-	-	+	+
AQ4299	O3:K6	+	-	-	-	-	-	-	+	+
KE10462	O3:K6	+	-	-	-	-	-	-	+	+
AQ4037	O3:K6	+	-	-	-	-	-	-	+	+
KE10461	O3:K6	+	-	-	-	-	-	-	+	+
KE9967	O3:K6	+	-	-	-	-	-	-	+	+
KE9984	O3:K6	+	-	-	-	-	-	-	+	+
U-5474	O3:K6	+	-	-	-	-	-	-	+	+
ATCC43996	O3:K4	+	-	-	-	-	-	-	+	+
UCM V553	O3:K53	+	-	-	-	-	-	-	+	+
UCM V493	O2:K28	+	-	-	-	-	-	-	+	+
30824	O4:K11	+	-	-	-	-	-	-	+	+
428/00	N/A	+	-	-	-	-	-	-	+	+
1324	O4:K8	+	-	-	-	-	-	-	+	+
